# Molecular and Immunohistochemical Expression of LTA4H and FXR1 in Canine Oral Melanoma

**DOI:** 10.3389/fvets.2021.767887

**Published:** 2021-12-13

**Authors:** Laura Nordio, Chiara Bazzocchi, Francesca Genova, Valentina Serra, Maria Longeri, Giovanni Franzo, Marco Rondena, Damiano Stefanello, Chiara Giudice

**Affiliations:** ^1^Department of Veterinary Medicine (DIMEVET), Università degli Studi di Milano, Milan, Italy; ^2^Department of Animal Medicine, Production and Health (MAPS), Università degli Studi di Padova, Legnaro, Italy; ^3^San Marco Veterinary Clinic and Laboratory, Veggiano, Italy

**Keywords:** dog, oral melanoma, LTA4H, FXR1, immunohistochemistry, prognostic markers

## Abstract

Oral melanoma is a common canine tumor whose prognosis is considered ominous, but poorly predicted by histology alone. In the present study the gene and protein expression of Leukotriene A4 hydrolase (LTA4H) and Fragile-X-mental retardation-related protein1 (FXR1), both reported as related to metastatic potential in different tumors, were investigated in canine oral melanoma. The main aim of the study was to confirm and quantify the presence of LTA4H and FXR1 genes and protein in oral melanomas. A secondary aim was to investigate their association with histologic prognostic criteria (mitotic count, Ki-67 index). Formalin-fixed-paraffin-embedded canine oral melanomas (36) were collected and histopathological evaluation carried out. Immunolabelling for LTA4H and FXR1 and Ki-67 were performed. RT-PCR evaluated LTA4H and FXR1 gene expressions. Histologically, most tumors were epithelioid cell melanomas (19/36) and were amelanotic, mildly or moderately pigmented (5, 12 and 13/36 respectively), only 6 were highly pigmented. Mitotic count ranged 1-106, Ki-67 index ranged 4.5–52.3. Thirty-two (32/32) melanomas immunolabelled for LTA4H and 33/34 for FXR1. RT-PCR values ranged 0.76–5.11 ΔCt for LTA4H and 0.22–6.24 ΔCt for FXR1. Molecular and immunohistochemical expression of both LTA4H and FXR1 did not statically correlate with mitotic count or Ki-67 index. The present study demonstrates LTA4H and FXR1 gene and protein in canine oral melanoma, however their expression is apparently unrelated to histopathologic prognostic criteria. Although LTA4H and FXR1 seem unrelated to tumor behavior, their extensive expression in the present cohort of cases suggest that they may play a role in canine oral melanoma oncogenesis.

## Introduction

Melanoma is a common neoplasia in dogs, that can be a diagnostic and prognostic challenge. Melanoma represents about 3% of all canine tumors and 7% of all canine malignant tumors. It originates in the oral cavity (62%), the skin (27%), the digit (6%) and, less commonly in the eye (5%) (1, 2). Melanoma is the most common malignant tumor of the oral cavity in dogs (3).

Oral melanomas are traditionally considered malignant, rapidly growing, invasive tumors that often recur after surgical resection and frequently metastasize, via lymphatic or blood vessels, to regional lymph nodes, lungs and viscera (2, 4). In dogs, the reported average survival time after diagnosis of oral melanoma spans 5–7 months (1, 5, 6). However, a subset of oral melanocytic tumors with a more favorable clinical course and prolonged survival have been reported, mainly in dogs with histologically well-differentiated melanocytic neoplasms (6–10). Several studies evaluated histological and immunohistochemical prognostic markers and possible threshold values to define morphological standards (6–8), but obtained different or even conflicting results. Currently, a parameter to effectively predict the likely progression of the disease in individual dogs is still not available.

Recent studies both in human and veterinary medicine have focused on finding genetic markers that may have a good predictive value, with specific emphasis on the potential development of metastatic disease (11–14). The studies of Onken and Malho investigated genetic biomarkers of uveal melanoma in humans and dogs, respectively (11–13). They highlighted the existence of different molecular classes of expected survival based on the differential expression of a set of genes. Among them, an increased expression in Leukotriene A4 hydrolase (LTA4H) and Fragile X mental retardation-related protein 1 (FXR1) genes were related to metastasizing behavior in both human and canine uveal melanoma.

LTA4H is a cytosolic hydrolytic enzyme, which catalyzes the conversion of leukotriene A4 into leukotriene B4 inside the arachidonic acid cascade. LTA4H expression is widely distributed in several different tissues (15). Leukotrienes are mediators of inflammation and chronic tissue inflammation has been linked to increased risk for the development of cancer (16). Aberrant arachidonic acid metabolism is suspected to have a role in carcinogenesis due to the imbalance shifted toward the pro-carcinogenic lipoxygenase pathways (5-, 8- and 12-LO) instead that anti-carcinogenic (15-LO) (16, 17). *LTA4H* has been previously described to be over-expressed in different types of tumors in humans, mice, rats, dogs and cats (18–28). *LTA4H* was also specifically demonstrated to be overexpressed in human, canine and feline ocular melanomas (10, 12, 28).

FXR1 is a cytoplasmic RNA binding protein, highly conserved among vertebrates and expressed in different tissues. FXR1 belongs to a family of RNA binding proteins consisting of the Fragile X Mental Retardation Protein (FMRP), responsible for the human fragile X mental retardation syndrome, and the Fragile X Mental Retardation Syndrome-Related Protein 2 (FXR2) (29, 30). FXR1 acts in the inflammatory process controlling the expression of tumor necrosis factor-α (TNF-α) at a post transcriptional level (31, 32) and has a role in muscular cells development (33). Moreover, its role has been investigated in different tumors such as squamous cell carcinoma (34), pulmonary carcinomas (35, 36), colorectal cancer (37), prostate cancer (38) and Wilms tumor (39). FXR1 is supposed to affect DNA stability with two pathways (40), either using miRNA pathway to regulate target mRNA expression (41) or playing a role in post-transcriptional regulation directly interacting with mRNA and affecting its stability (33). In lung tumorigenesis, for example, FXR1-dependent regulation of mRNA may specifically regulate ERK (extracellular-signal-regulated kinases) signaling pathway (36). Moreover, FXR1 recruits transcription factor STAT1 or STAT3 to gene promoters and, through the regulation of transcription, mediates cell proliferation in human cancers harboring TP53 homozygous deletion (42).

Our study aimed to investigate the expression of LTA4H and FXR1 in canine oral melanoma, at both genetic and protein level. A secondary aim of the study was to identify possible correlations between the expression of LTA4H/FXR1 and statistically proven histologic prognostic parameters, i.e., mitotic count and Ki-67 index.

## Materials and Methods

### Cases and Histopathology

Oral melanomas samples (*n* = 36) were collected and routinely formalin-fixed and paraffin-embedded (FFPE) (43) from the diagnostic service of histopathology at the University of Milan and the private San Marco Laboratory in Padova (Italy) during the period 2011–2017.

Four micrometer-thick sections of each sample were stained with hematoxylin and eosin (H&E) and evaluated on light microscope by two board certified pathologists (LN, CG). Mitotic count was calculated as the number of mitoses on 10 consecutive high power fields, starting in the area (0.237 mm^2^) with the highest mitotic activity (7).

Pigmentation of melanomas was semi-quantitatively evaluated on the whole section as: “negative/–” when no pigment was detectable, “mild/+” when 1 to 10% of neoplastic cells contain pigment, “moderate/++” 10 to 50% of cells were pigmented, and “high/+++” when >50% of cells were pigmented. In samples with no detectable pigment, the diagnosis of melanoma was confirmed through anti-PNL2 immunohistochemistry (see following section) (5, 44).

Additionally to the FFPEs, out of the 36, fresh samples were collected in double (two melanoma plus the adjacent not affected tissue) and stored in RNA later at −80°C for the subsequent molecular genetic analyses.

### Immunohistochemical Detection of LTA4H, FXR1, Ki-67, and PNL2

Serial paraffin sections were cut 4 μm thick and mounted on poly-lysine coated slides (Menzel-Gläser, Braunschweig, Germany). Immunohistochemical staining with the standard avidin-biotin-peroxidase complex (ABC) method was performed.

Briefly, sections were deparaffinized in xylene and rehydrated through a descending series of ethanol concentrations. Incubation with H_2_O_2_ 10% in Tris buffer saline (TBS; pH 7.4) for 2 h at 55°C in a laboratory stove was used to bleach heavily pigmented sections and to block endogenous peroxidase activity (45). In the set up phase of the protocol, selected samples of pigmented oral melanomas were treated with and without bleaching to assess if immunoreactivity was maintained.

Antigen retrieval was then performed by heating the slides in citrate buffer solution (pH 6.5) in a water bath at 95°C for 30 min (for LTA4H and FXR1) (46), in citrate buffer solution in pressure cooker for 18 min (for Ki-67) (47) and in microwave oven in EDTA buffer solution (pH 8.5) for 10 min at 500 W (for PNL2) (44), followed by cooling down in buffer at room temperature (RT) for 30 min. Sections were therefore incubated for 20 min at RT with normal horse (LTA4H, Ki-67, PNL2) or goat (FXR1) serum (dilution 1:70) to block any non-specific protein binding and therefore incubated with primary antibodies at 4°C overnight in a humidified chamber:

- mouse monoclonal leukotriene A4 hydrolase/LTA4H antibody (1E9 clone) (NBP1-47829; Novus Biologicals, Littleton, Co, USA), 1:100 dilution;
- rabbit polyclonal anti-FXR1 antibody (ab50841; Abcam, Cambdrige, UK), 1:100 dilution;
- mouse monoclonal anti-human Ki-67, clone MIB-1 (GA626; Dako, Glostrup, Denmark;), 1:600 dilution;
- mouse monoclonal anti Melanoma-Associated Antigen, clone PNL2 (MON3307; Monosan, Uden, Netherlands), 1:50 dilution.

Sections were then rinsed in TBS three times for three minutes each and incubated with the secondary anti-mouse (LTA4H; Ki-67; PNL2) or anti-rabbit (FXR1) biotinylated antibody (1:200, 30 min RT) (Vector Laboratories, Burlingame, CA, USA, BA 1000 and BA 2000, respectively) followed by rinsing in TBS and therefore incubation with the ABC reagent (30 minutes RT) (PK-6100; Vector Laboratories, Burlingame, USA). After rinsing in TBS, the 3-amino-9-ethylcarbazole (AEC) chromogen (SK-4200; Vector Laboratories, Burlingame, CA, USA) was applied for 15 min and, after rinsing in tap water, slides were counterstained with Mayer's haematoxylin (SS C030X; Diapath srl, Martinengo, Italy) for 2 min. Slides were therefore dried and mounted in aqueous mounting agent (108562; Aquatex, Merck, Darmstadt, Germany).

Canine skeletal myocytes adjacent to the neoplasm, and neutrophils were adopted as internal positive controls for FXR1 and LTA4H immunolabelling, respectively (46, 48). Epithelium of intestinal crypts in a section of normal canine intestine was adopted as positive control for Ki-67, a section of partially pigmented canine melanoma was adopted as positive control for PNL2. Negative controls were carried out by replacing the primary antibodies with mouse or rabbit IgG (Santa Cruz; Dallas, TX, sc-2025 and sc2027, respectively).

Immunolabelled sections were evaluated at optic microscope. LTA4H and FXR1 labeled sections were semi-quantitatively scored, considering the percentage of positive cells (<10, 11–30, 31–50, 51–70, >70%), cellular localization of the signal (nuclear/cytoplasmic), intensity of staining (mild, moderate, intense). The immunoreactive score adapted from Remmele and Stegner (IRS score) (49) was calculated combining the intensity and percentage of positivity as follows: IRS score (0–12) = percentage of positive cells (no positive cells = 0, 1–10% positive cells= 1, 11–50% of positive cells= 2, 51–70% of positive cells= 3, >70% of positive cells = 4) ^*^ intensity of staining (no positive cells = 0, +/mild = 1, 2+/moderate = 2, 3+/marked = 3). For statistical purposes, IRS scores were further categorized into three cumulative classes of expression, as follows: class 1 (mild expression) corresponded to IRS scores 0–3, class 2 (intermediate expression) to IRS scores 4–8 and class 3 (marked expression) to IRS scores 9–12.

Ki-67 index was calculated as the mean number of positively labeled neoplastic cell nuclei in 5 fields at 400× (7). The number of positive nuclei was counted using a digital automatic counter (QCapture Pro 6.0). PNL2 positivity was evaluated as presence or absence of the signal.

### Relative Expression of *LTA4H* and *FXR1*

Total RNA was extracted from eight sections 10 μm-thick cut from each FFPE tissue block using the RecoverAll Total Nucleic Acid Isolation Kit (AM1975; Thermofisher Scientific; Massachusetts, USA) and from frozen fresh samples using RNeasy Minikit (74104; Qiagen; Hilden, Germany). RNA was eluted in a final volume of 60 μl of water after an on-column DNase treatment (Qiagen; Hilden, Germany), quantified using NanoDrop ND-100 Spectrophotometer (Thermofisher Scientific; Massachusetts, USA) and immediately stored at −80°C until molecular analyses.

Two hundred nanograms of RNA were retro-transcribed to cDNA using the QuantiTect Reverse Transcription kit (205311; Qiagen; Hilden, Germany) following the manufacturer's protocol. An additional reaction without retrotranscriptase enzyme was performed to verify the complete DNA removal. cDNAs were stored at −80°C until subsequent use.

Amplification condition, the dynamic range and the efficiency of each qPCR reaction were assessed on cDNA produced from a fresh tissues. Furthermore, the integrity of the RNA extracted from FFPE samples was verified through the amplification of a fragment of *B2M* gene from all cDNA.

The relative expression of *LTA4H* and *FXR1* genes for each FFPE was calculated after a ΔCt measure using the *B2M* and *ACTB* genes as references. The synthesized cDNA samples were amplified in duplicate using the iQ5 Real-Time PCR instrument (Bio-Rad, California, USA) and Universal SYBR^®^ Green Supermix (1708880; Bio-Rad, California, USA) as fluorescent molecules. Primers sequences were described by Malho and coauthors (11) and the final concentration of forward and reverse primers was 250 nM for *FXR1, B2M* and *ACTB* and 400 nM for *LTA4H* genes, respectively (Supplementary Table 2). The thermal profile was 98°C for 30 s, 40 cycles of 98°C for 15 s, 50–58°C for 15 s, 72°C for 15 s and a melting profile was included after the last amplification cycle in order to exclude the presence of aspecific amplifications. Cycle threshold (Ct) values of each target were determined for each sample to estimate ΔCt measure.

### Statistical Analysis

Molecular and immunohistochemical levels of LTA4H and FXR1 were compared between tumors with low (<19.5) and high Ki-67 index (≥19.5) and tumors with low (<4) and high (≥4) mitotic count, according to the prognostic thresholds proposed by Bergin and colleagues (7). Adopted statistical tests were the Mann Whitney U test and the Kruskal-Wallis test (*p* < 0.05). Correlation between immunohistochemical IRS scores and molecular ΔCt was assessed by the Spearman Rho test (*p* < 0.05).

## Results

### Case Selection and Histology

Thirty-six specimens of oral melanomas from 32 dogs were included in the study (two samples were recurrence and one was a necropsy sample of cases already included in the study, and one dog had two distinct masses in different sites of the oral cavity). Dogs belonged to different breeds and ranged 5–18 years of age (mean 11.8, median 12). Fifteen animals were male, fifteen were female, and for two animals sex was not reported. When the specific site of the tumor was provided, oral melanomas were located in the gum (*n* = 10, 27.8%), labial mucosa (*n* = 5, 13.9%), palate (*n* = 2, 5.6%) and tonsillary region (*n* = 1, 2.8%).

Histologically, melanomas exhibited different morphological types (epithelioid, spindle or mixed) and various degree of pigmentation. Nineteen tumors (52.8%) were classified as epithelioid, nine (25%) as spindle and eight (22.2%) as mixed. Pigmentation was evaluated as absent in 5 tumors (13.9%), mild/+ in 12 (33.3%), moderate/++ in 13 (36.1%) and high/+++ in 6 (16.7%). Mitotic count ranged 1–106 (mean 23.8, median 15). Five tumors had a mitotic count <4, whereas 31 had mitotic count ≥4 (Supplementary Table 1).

### Immunohistochemical Expression of LTA4H, FXR1 and Ki-67

Of the originally selected 36 cases, 2 cases were excluded from the immunohistochemical results because they were poorly reactive, and 2 cases were excluded from the anti-LTA4H staining because no further material was available in the paraffin block. Immunoreactivity was of equal intensity in the preliminary subset of samples treated with and without bleaching, confirming the maintenance of immunoreactivity after the bleaching protocol (Supplementary Table 1).

**Figure 1:**
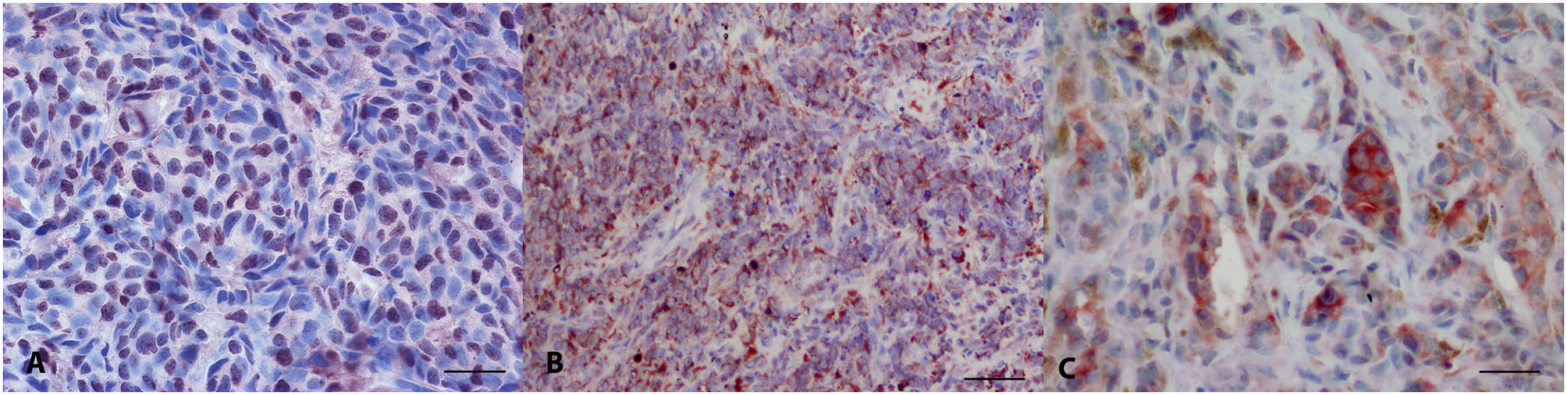
Canine oral melanoma, immunohistochemistry. **(A)** Anti-LTA4H staining, diffuse nuclear signal. AEC chromogen, bar 50 μm. **(B)** Anti-LTA4H staining, diffuse cytoplasmic signal. AEC chromogen, bar 100 μm. **(C)** Anti-FXR1 multifocal intense signal. AEC chromogen, bar 50 μm.

Thirty-two melanomas were LTA4H-positive (32/32) (Figures 1A,B), with a percentage of positive neoplastic cells of 31–50% in 2 cases, 51–70% in five and above 70% in 25 cases. The intensity of staining was from mild to intense. The localization of the positivity was cytoplasmic (*n* = 19), nuclear (*n* = 3), or both nuclear and cytoplasmic (*n* = 10). IRS score varied from 3 to 12. 18/32 cases (56.3%) were classified as IRS score class 2, 14/32 cases (43.8%) as class 3.

FXR1 immunolabelling stained positive in 33/34 tested oral melanomas (Figure 1C). The percentage of stained cells was <10% in 6 cases, between 11 and 30% in 5 cases, between 51 and 70% in 9 and >70% in 8 cases. Fourteen cases were intensely stained, 13 moderately and 6 mildly. The immunostaining was always cytoplasmic. IRS score varied from 0 to 12. 6/34 cases (17.6%) were classified as IRS score class 1, 13/34 cases (38.2%) as class 2 and 11/34 cases (32.4%) as class 3.

Ki-67 index was assessed in 28/34 cases, due to insufficient paraffin block material. The average number of positive neoplastic nuclei ranged 4.5–52.3, with a mean of 16.9 and a median of 13.7. Ki-67 index was beneath 19.5 in 19 cases and above 19.5 in 9 cases.

The comparison of groups with low and high Ki-67 index (<19.5 and ≥19.5) with the levels of immunohistochemical scores of LTA4H and FXR1 revealed no statistically significant differences (*p* = 0.212 and 0.138, respectively). Groups with low and high mitotic count (<4 and ≥4) had no significantly different levels of immunohistochemical FXR1 (*p* = 0.153) but had significantly different IRS scores of LTA4H (*p* = 0.017). The intracellular localization of LTA4H immunohistochemical signal (nuclear, cytoplasmic, or nuclear and cytoplasmic) showed no statistical correlation with LTA4H IRS score (*p* = 0.532).

### *LTA4H* and *FXR1* Gene Expression

Thirty-six FFPE cases of canine oral melanoma were analyzed with qPCR to quantify the expression of the target genes *LTA4H* and *FXR1* (Supplementary Table 1).

*B2M* and *ACTB* were preliminary tested as housekeeping genes in a subset of 10 samples, to assess if the expression trend was stable. Despite the presence of a difference in the threshold cycles (Ct) of *B2M* and *ACTB* (paired *t*-test: *t*9 = 8.48; *p* < 0.0001), the relation between the two genes expression was linear (*p* < 0.0001), thus indicating that the ratio of expression between *B2M* and *ACTB* was constant. The Ct of these two housekeeping genes were related by the equation:


$$\begin{array}{l} Ct\;B2M\;=7.3+.82^{*}Ct\;ACTB\;\left(R2\;=\;0.92\right). \end{array}$$

Therefore, *B2M* was chosen as housekeeping gene to complete the analyses of the samples in order to quantify the relative expression of the investigated target genes.

Among the analyzed samples, RNA extraction suitable for cDNA synthesis and qPCR assays was obtained in twenty-three out of thirty-six cases and in these samples *LTA4H* and *FXR1* were successfully amplified. Gene expression results, reported as ΔCt, comparing the Ct of the target gene with the Ct of the housekeeping gene, were ranged 0.76–5.11 for *LTA4H* and 0.22–6.24 for *FXR1*. In three samples the amplification of *FXR1* gene was not quantified due to the Ct value being out of the dynamic range of the reaction.

**Figure 2:**
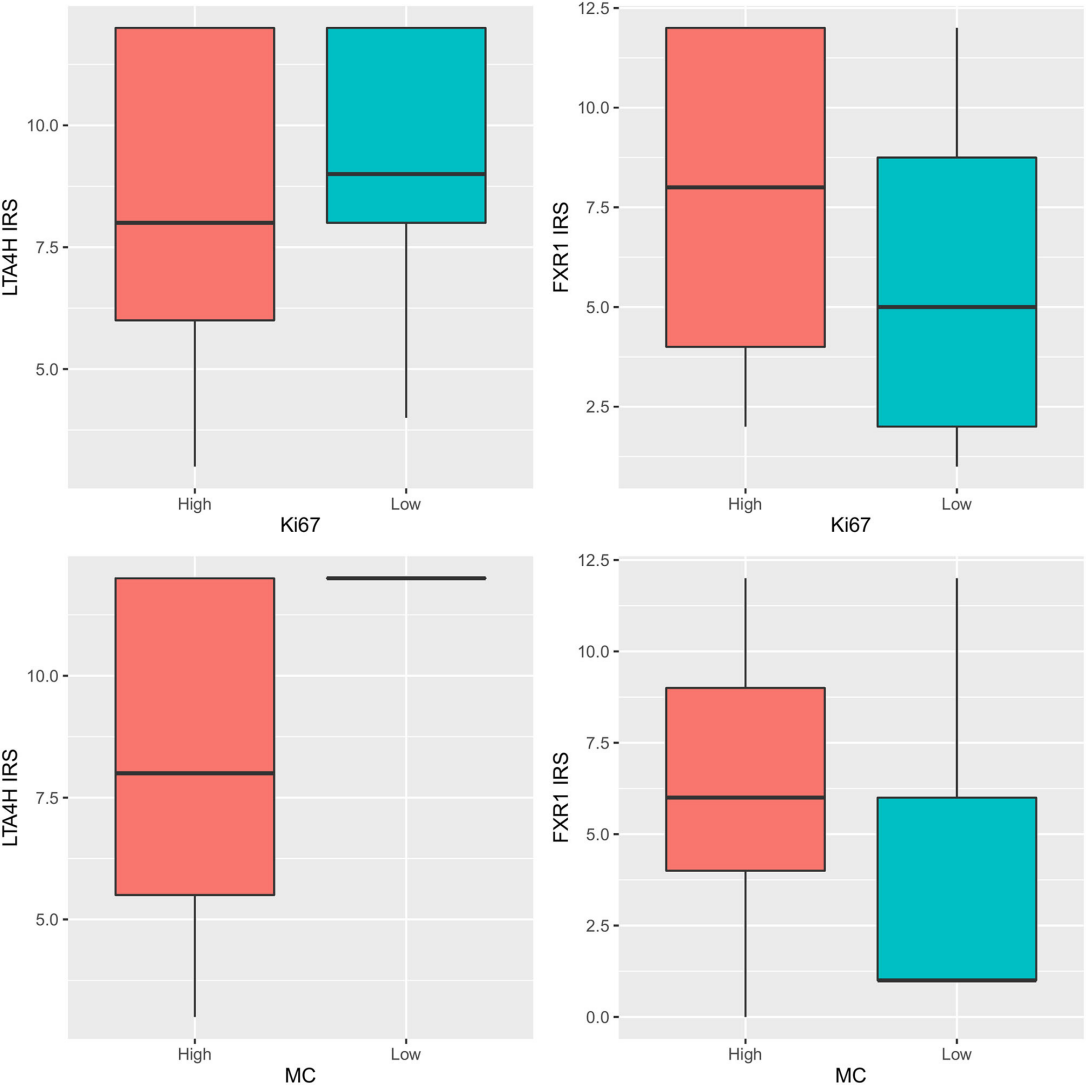
LTA4H and FXR1 IRS scores: comparison among groups with low and high Ki-67 index (upper row) (upper left *p* = 0.212, upper right *p* = 0.138) and among groups with low and high mitotic count (MC) (lower row) (lower left *p* = 0.017, lower right *p* = 0.153).

Molecular expression of both *LTA4H* and *FXR1* genes did not differ among groups with low and high Ki-67 index (<19.5 and ≥19.5) (*LTA4H p* = 0.502, *FXR1 p* = 0.635), nor among groups with low and high mitotic count (<4 and ≥4) (*LTA4H p* = 0.501, *FXR1 p* = 0.140). *LTA4H* molecular and immunohistochemical values were correlated (*p* = 0.014, rho = −0.539), but *FXR1* was not (*p* = 0.122, rho = 0.337) (Figure 2).

## Discussion

In the present study, a caseload of canine oral melanomas was investigated for the expression of two targets, i.e., LTA4H and FXR1, at both gene and protein level.

Anamnestic and histological data of dogs included in the study were mostly consistent with previous literature reports: dogs were equally distributed among sexes and variably distributed among breeds, with a preponderance of mixed breed. Adult-old aged dogs were most represented (mean 11.8 years of age) (7, 50) and tumors mainly affected the gum (2). Consistently with literature (1–5, 8), neoplastic cell histo-morphology was highly variable, with epithelioid cell type predominantly observed, and in the vast majority of cases <50% of neoplastic cells were pigmented Although a “high” degree of pigmentation (at least 50% of pigmented cells) has been correlated with longer survival times in canine oral melanomas, the prognostic significance of the degree of tumor pigmentation is still debated, most likely due to the subjectivity in the quantification of the pigment (7, 8).

The main aim of the present study was to investigate the occurrence of LTA4H and FXR1 genes and proteins in canine oral melanoma. *LTA4H* and *FXR1* have been previously found to be over-expressed in both human and canine uveal melanomas (11, 12, 14) and *LTA4H* has also been reported to be upregulated in feline ocular melanomas (28). Additionally, the immunohistochemical expression of FXR1 protein has been demonstrated in a small caseload of canine uveal and oral melanocytic tumors (46).

In the present cohort of cases, LTA4H and FXR1 were detected at both gene and protein levels. The qPCR relative expression of *LTA4H* and *FXR1* genes exhibited marked differences among tested cases of oral melanoma, conversely immunohistochemical positivity showed negligible expression differences. Comparing immunohistochemical results, expressed as IRS score, with qPCR results, expressed as ΔCt, a correlation among protein and gene expression was significant only for LTA4H. This discrepancy between immunohistochemical and qPCR results can have different explanations. First, immunohistochemistry, although useful in providing information concerning the localization of antigens, is not a quantitative technique, as opposite to RT-PCR. Second, protein tissue expression does not instantly reflect possible alterations during the synthesis process, which can take place from transcription to post-translational modifications, i.e., protein expression does not necessarily mirror gene expression. Third, while the use of FFPE tissues allows to enroll archive specimens, formalin ficxation procedure can induce degradation of the tested molecules. However, in the present caseload, DNA quality was assessed through the amplification of house-keeping genes and only samples in which the RNA extraction was considered suitable for cDNA synthesis and qPCR assays were further included in the study. Finally, molecular analyses may be influenced by residual constitutive expression of genes in normal adjacent tissues or extracellular matrix.

The secondary aim of the present study was to evaluate the possible association LTA4H and FXR1 genes and proteins with established histologic prognostic criteria, i.e., mitotic count and Ki-67 index (7, 42), in order to test if LTA4H and FXR1 presence and level of expression could be related to the biological behavior of the tumor.

Data on the expression of the investigated targets were compared with mitotic count, and no difference between melanomas with mitotic count < or > 4/10 HPF (8) was observed for FXR1 neither at gene nor at protein levels, while a significantly difference was observed for LTA4H protein but not gene expression No statistically significant association was observed between LTA4H and FXR1 when their gene and protein expressions were compared with Ki-67 index (adopting a value of 19.5 as a discriminating threshold) (7).

Overall, the results of this study indicate that LTA4H and FXR1 are extensively expressed in canine oral melanomas and suggest that they can play a role in tumor oncogenesis process. However, their gene and protein expressions resulted poorly related to histological prognostic criteria suggesting that they are not associated to canine oral melanoma biological behavior, conversely to what has been demonstrated in canine and human uveal melanomas.

It should be noted that, in previous studies, the overexpression of *LTA4H* and *FXR1* genes has been specifically associated with increased metastatic risk of uveal melanoma, while in the present study LTA4H and FXR1 were compared with histopathologic prognostic criteria, but not specifically with the presence of metastases. In this regard, it is worth considering that canine uveal and oral melanocytic tumors are characterized by different biological behavior. Indeed, while uveal melanomas are generally slowly progressive and slowly metastasizing tumors, oral melanomas are aggressive tumors with rapid progression and frequently already metastasized at the time of diagnosis (2, 4, 51, 52). Therefore, the more homogeneous, rapidly aggressive, behavior of canine oral melanomas enrolled in the study may affect the lack of discriminating value of the markers that we investigated.

In conclusion, in the present study the expression of LTA4H and FXR1 has been verified in canine oral melanoma at both gene and protein levels, and even though we failed to identify a significant correlation with known histopathologic criteria of prognosis, further investigations on a larger cohort of cases and possibly including follow up data are necessary to define the role of FXR1 and LTA4H in the pathogenesis of canine oral melanoma.

## Data Availability Statement

The original contributions presented in the study are included in the article/Supplementary Material, further inquiries can be directed to the corresponding author.

## Ethics Statement

Ethical review and approval was not required for the animal study because this study did not include experimental animals, it was performed on histological specimens. All animal owners signed an informed consensus for clinical procedures and data recording and using for studies.

## Author Contributions

LN and CG performed the histology and immunohistochemistry. LN, CG, and CB wrote the first draft of the manuscript. MR and DS contributed to case selection. CB, FG, VS, and ML performed the molecular analyses. GF performed the statistical analysis. All authors contributed to manuscript revision, read, and approved the submitted version.

## Funding

The authors acknowledge support from the University of Milan through the APC initiative.

## Conflict of Interest

The authors declare that the research was conducted in the absence of any commercial or financial relationships that could be construed as a potential conflict of interest.

## Publisher's Note

All claims expressed in this article are solely those of the authors and do not necessarily represent those of their affiliated organizations, or those of the publisher, the editors and the reviewers. Any product that may be evaluated in this article, or claim that may be made by its manufacturer, is not guaranteed or endorsed by the publisher.
