# Supplementary material for: Molecular and Immunohistochemical Expression of LTA4H and FXR1 in Canine Oral Melanoma
Source: Front Vet Sci. 2021 Dec 13;8:767887. doi: 10.3389/fvets.2021.767887 (PMC8710725; doi:10.3389/fvets.2021.767887)
Supplement: Supplementary file 1 [file Data_Sheet_1.PDF]

TABLE 1 SUPPLEMENTAL

SUMMARY OF CASELOAD OF ANALYZED CANINE ORAL MELANOMAS, WITH SIGNALMENT, HISTOLOGY, IHC, Q-PCR.

									IHC LTA4H		IHC FXR1		q-PCR	
	Breed	sex	Age	Oral cavity: location	Cell type	Pigment	MC	Ki67 index	localization	IRS score	localization	IRS score	LTA4H ΔCt	FXR1 ΔCt
1	Bernese mountain dog	SF	11	labial mucosa	spindle	+++	5	na	na	na	negative	0	na	na
2	English setter	M	15	na	epithelioid	+	71	13,33	c + n	12	c	12	1.34	3,57
3	Dachshund	M	na	gum, upper maxillary arch	spindle	+	28	10,00	c	8	c	4	3,46	2,13
4	Mixed breed	M	10	na	spindle	+	39	7,08	n	12	na	na	4,14	5,42
5	Pit bull	F	13	upper labial mucosa	epithelioid	++	8	12,63	c	12	c	4	3,4	3,45
6	Shar pei	SF	7	palate	spindle	+	18	4.50	c	4	c	9	3,23	5,86
7	Mixed breed	SF	12	na	epithelioid	++	17	22,33	c + n	3	c	6	4,7	5,86
8	Mixed breed	SF	18	na	epithelioid	+++	37	27,21	c + n	12	c	2	na	na
9	Dachshund	M	14	gum	epithelioid	+	11	11,67	c	8	c	6	3,07	5,55
10	Mixed breed	SF	12	gum/ labial mucosa	spindle	+	54	12,08	c	12	c	9	3,89	4,48
11	Rottweiler	SF	7	na	mixed	-	43	33,33	c + n	12	c	12	0.76	0,92
12	Golden retriever	F	14	na	mixed	+++	2	5,71	c	12	c	1	na	na
13	Nd	nd	nd	labial mucosa	epithelioid	++	7	15,00	c	4	c	6	4,37	6.24
14	Mixed breed	SF	13	gum	spindle	+++	1	5,33	c + n	12	c	6	3,93	out of range (>35)
15	Mixed breed	NM	14	na	epithelioid	++	11	5,54	c	8	c	9	na	na
16	English setter	SF	10	soft palate	epithelioid	+++	3	18,17	c	12	c	1	na	na
17	Mixed breed	M	14	na	mixed	-	11	39,42	c	6	c	12	4,17	5,12
18	Nd	M	14	na	epithelioid	++	5	7,79	c	8	c	2	na	na
19	Mixed breed	M	11	na	spindle	++	9	na	na	na	c	6	4,09	out of range (>35)
20	German sheperd	M	11	gum	epithelioid	++	17	24,42	c	8	c	4	na	na
21	West highland white terrier	SF	7	gum	mixed	++	26	19,67	c + n	6	c	12	2,56	4,6
22	German sheperd	M	16	na	mixed	++	7	17,67	c	12	c	2	2,05	3,44
23	Dachshund	M	11	gum	epithelioid	+++	2	11,67	c + n	12	c	1	na	na
24	Mixed breed	nd	nd	na	epithelioid	++	8	na	c	4	c	2	na	na
25	Mixed breed	SF	12	oral vestibule	epithelioid	++	92	52,25	c	4	c	2	na	na
26	Mixed breed	F	11	tonsillar region	epithelioid	+	27	14	c	8	c	4	4	0.22

27	Hungarian spitz	M	13	maxillary region	epithelioid	+	28	na	n	4	c	6	na	na
28	Labrador retriever	NM	13	gum	epithelioid	+	6	na	na	na	na	na	3,66	5,38
29	Mixed breed	F	10	gum, maxillary area	spindle	+	6	na	c	4	c	9	5.11	out of range (>35)
30	Pekingese	M	12	gum, molar area	epithelioid	++	44	10,33	c	8	c	2	4,05	3,34
31	Golden retriever	F	5	gum	epithelioid	++	1	na	na	na	c	12	4,41	5,94
32	German sheperd	M	11	oral cavity mandible region	mixed	+	22	na	c + n	9	c	6	3,99	3,33
33	German sheperd	M	11	oral cavity mandible region	mixed	+	13	10,42	c + n	9	c	9	na	na
34	Mixed breed	M	12	lower lip mucosa	epithelioid	-	49	23,08	c	12	c	8	na	na
35	Beagle	M	14	upper gum	mixed	-	22	22,25	c + n	8	c	12	4,45	4,31
36	Beagle	M	14	upper gum (necropsy of #36)	mixed	-	106	15,92	n	6	c	8	4,04	3,85

Legend: MC= Mitotc count, c= cytoplasmic, n= nuclear, na = not assessed; 1 = mild, 2 = moderate, 3 = intense.

TABLE 2 SUPPLEMENTAL

PRIMERS SEQUENCES AND AMPLIFICATION FRAGMENT SIZE

Name	Sequence	Amplification fragment size
LTA4H F	5’ GTCACTCTCCAATGTTATTG 3’	73 base pairs
LTA4H R	5’ CCAAGTTTTGTTGGTCAC 3’	
FXR1 F	5’ TGGCAATTGGAACACATG 3’	115 base pairs
FXR1 R	5’ TCAGCACTCTCTCCATAG 3’	
B2M F	5’ TCCTCATCCTCCTCGCT 3’	85 base pairs
B2M R	5’ TTCTCTGCTGGGTGTCG 3’	
ACT F	5’ ATCGCTGACAGGATGCAGAA 3’	141 base pairs
ACT R	5’ ACATTTGCTGGAAGGTGGACA 3’	
