# Supplementary material for: Molecular and Immunohistochemical Expression of LTA4H and FXR1 in Canine Oral Melanoma
Source: Front Vet Sci. 2021 Dec 13;8:767887. doi: 10.3389/fvets.2021.767887 (PMC8710725; doi:10.3389/fvets.2021.767887)
Supplement: Supplementary file 2 [file Data_Sheet_2.PDF]

Figure S1: Canine tissues, immunohistochemistry, AEC chromogen. A) Anti- FXR1 staining, positive control: diffuse intense cytoplasmic signal of muscle fibers. Bar 50 µm. B) Anti-LTA4H staining, diffuse nuclear positive staining of neutrophils on the surface of an ulcerated tumor. Bar 50 µm.

Figure S2: Canine oral melanoma, immunohistochemistry, AEC chromogen. A) Anti- FXR1 staining, negative control. Bar 50 µm. B) Anti-LTA4H staining, negative control. Bar 50 µm.

Figure S3: Canine tissues, immunohistochemistry, AEC chromogen. A) Canine oral melanoma: Anti-KI67 staining, intense, diffuse nuclear positive staining of neoplastic cells. Bar 50 µm. B) Canine intestine (puppy): Anti- KI67 staining, positive control. Intense, diffuse nuclear positive staining of epithelial cells. Bar 50 µm.

Figure S4: Canine oral melanoma, immunohistochemistry, AEC chromogen. A) Anti- PNL2 staining, diffuse, intense cytoplasmic staining control. Bar 50 µm. B) Anti-LTA4H staining, negative control. Bar 50 µm.
