# Supplementary figures and images for: Molecular and Immunohistochemical Expression of LTA4H and FXR1 in Canine Oral Melanoma
Source: Front Vet Sci. 2021 Dec 13;8:767887. doi: 10.3389/fvets.2021.767887 (PMC8710725; doi:10.3389/fvets.2021.767887)

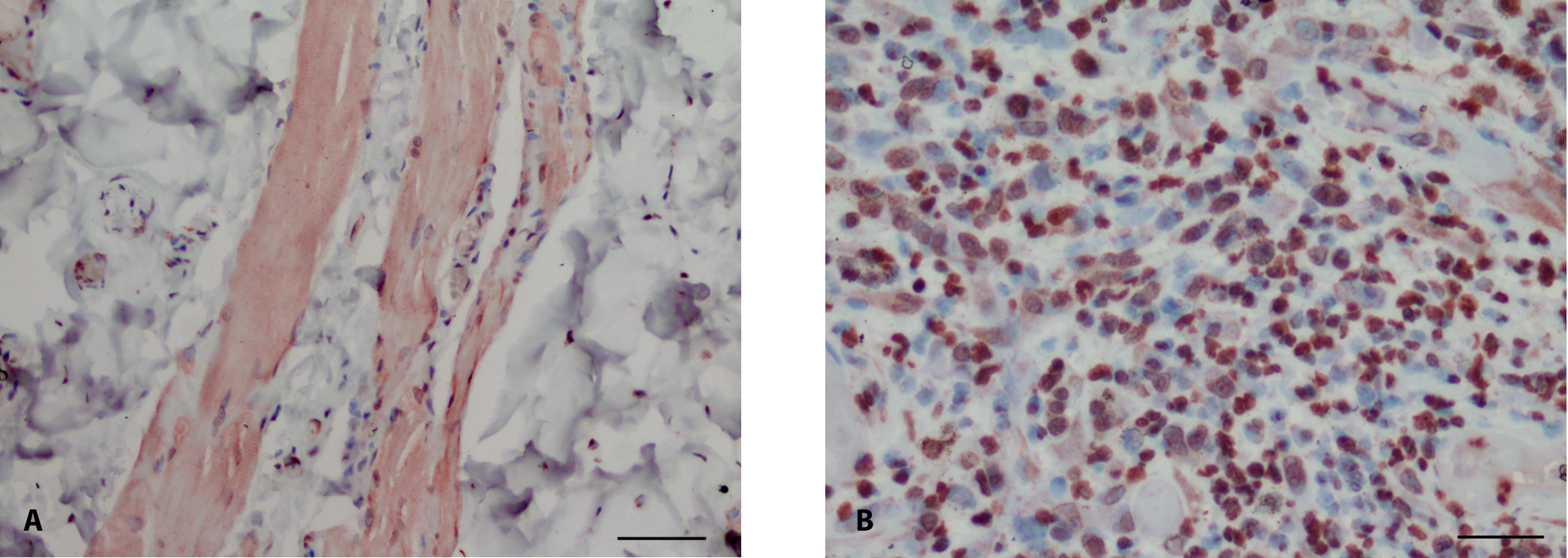

Supplement: Supplementary file 3 [file Image_1.tif]

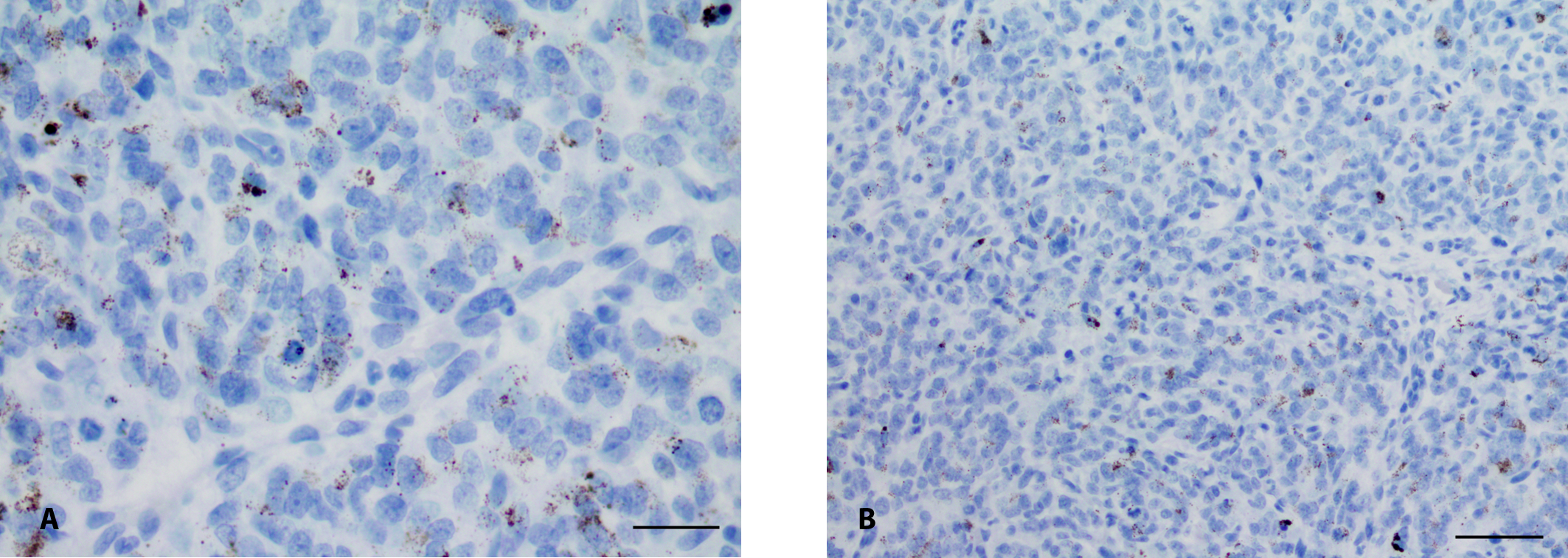

Supplement: Supplementary file 4 [file Image_2.tif]

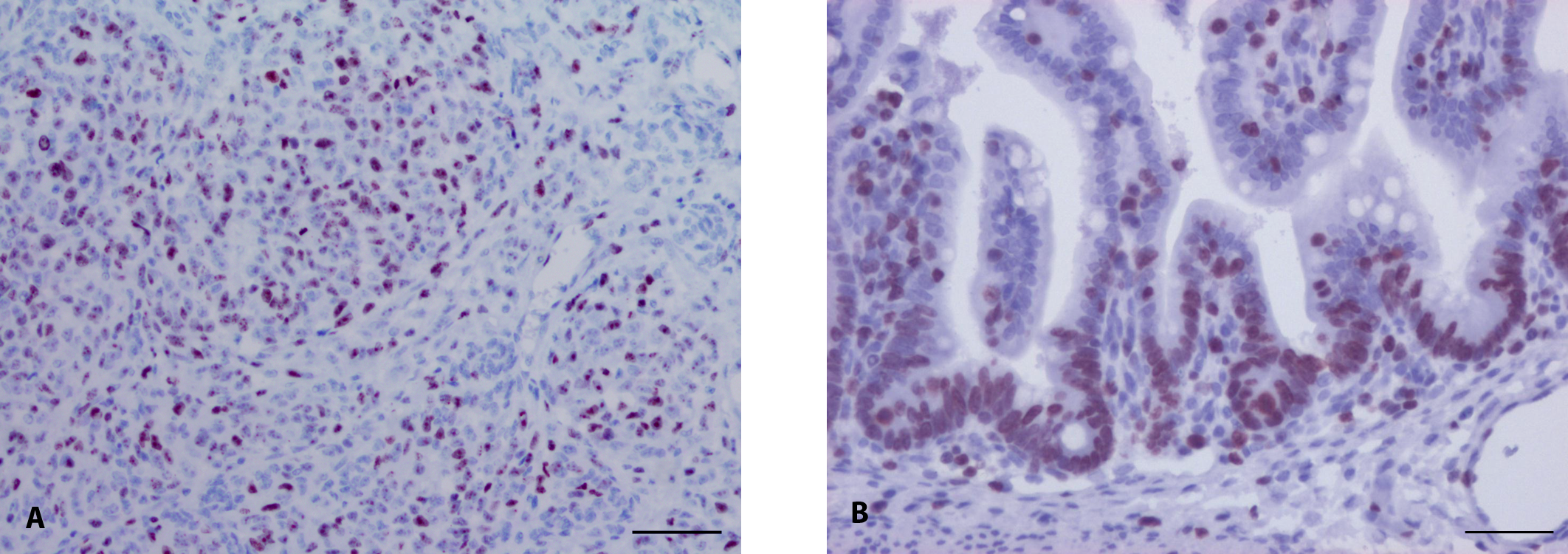

Supplement: Supplementary file 5 [file Image_3.tif]

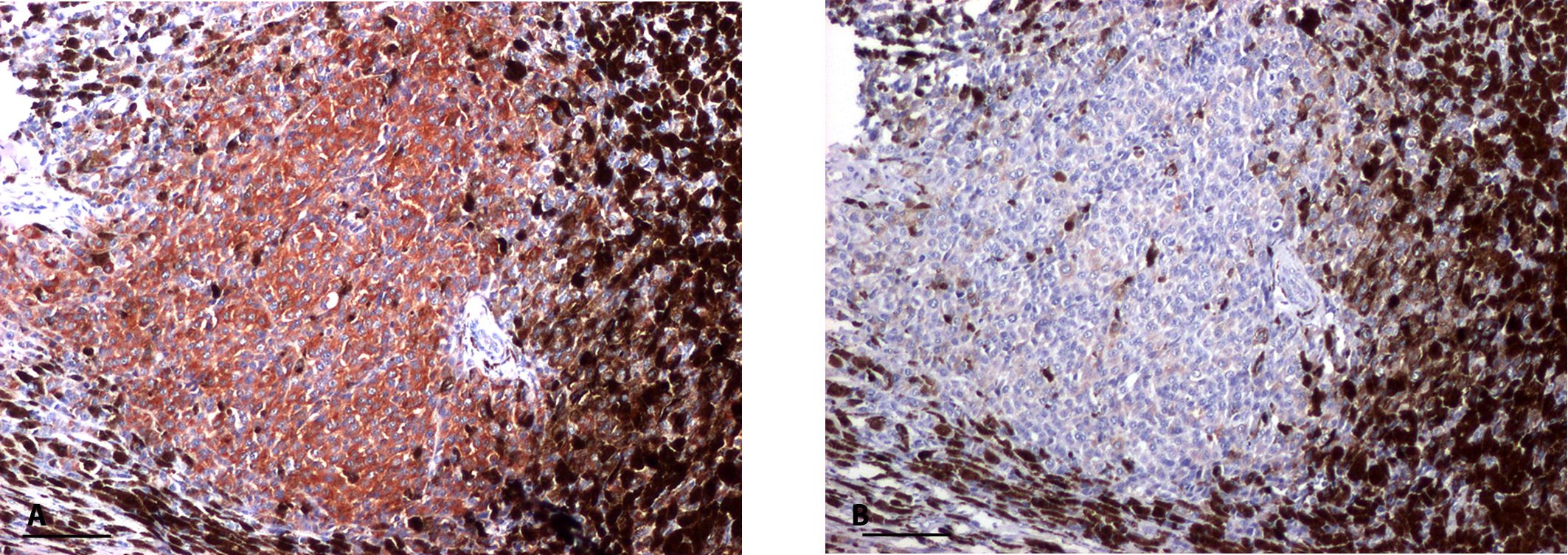

Supplement: Supplementary file 6 [file Image_4.tif]
